# Optimizing Encapsulation: Comparative Analysis of Spray-Drying and Freeze-Drying for Sustainable Recovery of Bioactive Compounds from *Citrus x paradisi* L. Peels

**DOI:** 10.3390/ph17050596

**Published:** 2024-05-07

**Authors:** Jolita Stabrauskiene, Lauryna Pudziuvelyte, Jurga Bernatoniene

**Affiliations:** 1 Department of Drug Technology and Social Pharmacy, Lithuanian University of Health Sciences, LT-50161 Kaunas, Lithuania; jolita.stabrauskiene@lsmu.lt (J.S.); lauryna.pudziuvelyte@lsmu.lt (L.P.); 2 Institute of Pharmaceutical Technologies, Lithuanian University of Health Sciences, LT-50161 Kaunas, Lithuania

**Keywords:** encapsulation, spray-drying, freeze-drying, *Citrus x paradisi* L. peels

## Abstract

Spray-drying and freeze-drying are indispensable techniques for microencapsulating biologically active compounds, crucial for enhancing their bioavailability and stability while protecting them from environmental degradation. This study evaluates the effectiveness of these methods in encapsulating *Citrus x paradisi* L. (grapefruit) peel extract, focusing on sustainable recovery from waste peels. Key objectives included identifying optimal wall materials and assessing each encapsulation technique’s impact on microencapsulation. The investigation highlighted that the choice of wall material composition significantly affects the microencapsulation’s efficiency and morphological characteristics. A wall material mixture of 17 g maltodextrin, 0.5 g carboxymethylcellulose, and 2.5 g β-cyclodextrin was optimal for spray drying. This combination resulted in a sample with a wettability time of 1170 (s), a high encapsulation efficiency of 91.41%, a solubility of 60.21%, and a low moisture content of 5.1 ± 0.255%. These properties indicate that spray-drying, particularly with this specific wall material composition, offers a durable structure and can be conducive to prolonged release. Conversely, varying the precise compositions used in the freeze-drying process yielded different results: quick wettability at 132.6 (s), a solubility profile of 61.58%, a moisture content of 5.07%, and a high encapsulation efficiency of 78.38%. The use of the lyophilization technique with this latter wall material formula resulted in a more porous structure, which may facilitate a more immediate release of encapsulated compounds and lower encapsulation efficiency.

## 1. Introduction

Phenolic compounds such as flavanones naringin and naringenin are abundant bioactive components in *Citrus x paradisi* L (grapefruit) [1]. These compounds have attracted the scientific community’s attention due to their unique health-promoting properties and diverse biological activities [2,3]. However, limited solubility, bioavailability, and stability often hinder their practical application in the pharmaceutical and food industries [4,5].

Microencapsulation technology is used in various industries, including food, cosmetics, and pharmaceuticals, to protect, isolate, and control the release of bioactive substances [6]. Microcapsules are particles of an outer shell enclosing an inner core containing the active ingredient (Table 1). The particle size of microcapsules can vary from 0.2 to 5000 μm, depending on the materials and processing methods used [7,8]. Methods such as spray drying, cooling, extrusion coating, liquid layer coating, liposome entrapment, lyophilization, coacervation, centrifugal suspension separation, crystallization, and inclusion complexation can produce microcapsules [9].

Spray-drying is widely used for converting liquid extracts into powder form [15]. It is practical and universal and can preserve phenolic compounds’ stability, solubility, and controlled-release characteristics [16]. Spray-drying also allows for the creation of microcapsules with different particle sizes, which can be customized to meet specific usage and formulation requirements, storage conditions, and desired shelf life [17,18,19].

**Figure 1:**
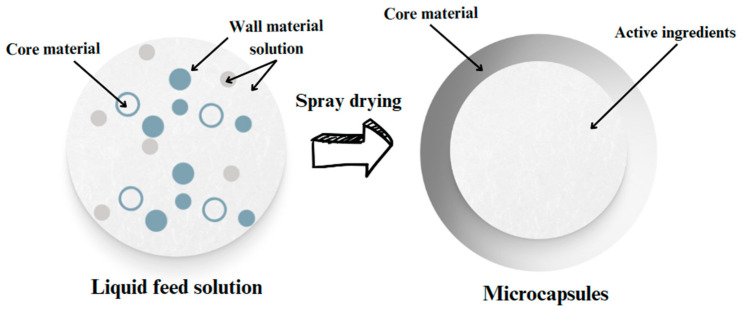
This schematic illustrates the encapsulation process, where a shell is formed around particles during the spray-drying procedure.

Before spray-drying, it is essential to emulsify the liquid extracts. Emulsification significantly influences the encapsulation efficiency, powder attributes, and stored materials’ stability [20,21] (Figure 1).

The stability of the emulsions, critical for effective microencapsulation, largely depends on the pH level. The acidic conditions (pH 3.0–6.0) are optimal for stabilizing grapefruit extracts rich in flavonoids and ascorbic acid; the latter is particularly vulnerable to degradation at higher pH levels [22]. Excipients such as β-cyclodextrin and carboxymethylcellulose enhance the solubility and stability of these bioactive compounds under acidic conditions, playing pivotal roles in maintaining the integrity of the emulsion [10,23].

**Figure 2:**
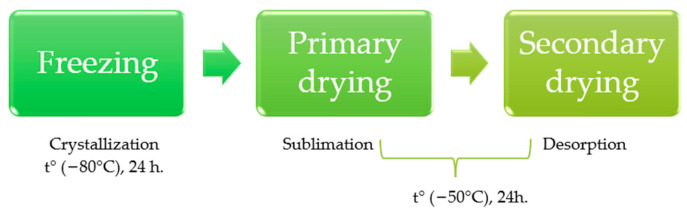
An overview of the three stages involved in the freeze-drying process.

Freeze-drying–lyophilization is a method of dehydration that delicately removes the solvent from a sample through sublimation during the primary drying phase and desorption during the secondary drying phase [24]. The process comprises three stages. The first is sample freezing; in this phase, the sample is frozen quickly to solidify the solvent and maintain the material’s structural integrity. This step is crucial in stabilizing the sample in a fixed geometry, which helps retain the sample’s physical structure during drying. During the primary drying (sublimation) phase, the solid solvent (such as water) transitions directly from the solid phase (ice) to the vapor phase under a high vacuum without passing through the liquid phase [25]. This phase is responsible for most of the solvent removal, and controlled heat is applied cautiously to provide the energy required for sublimation while preserving the integrity of the temperature-sensitive materials. In the secondary drying (desorption) phase, residual solvent, usually bound to the product, is removed by desorption (Figure 2). The temperature is raised above the primary drying levels to break the intermolecular forces holding the solvent molecules, helping to release them from the sample matrix. This phase ensures the complete removal of water, resulting in a dry product with an extended shelf life [26,27].

Freeze-drying is widely recognized as the best method for encapsulating sensitive bioactive compounds due to its gentle dehydration process, one which does not expose substances to high temperatures, unlike spray-drying [24]. This technique, known for its simplicity and quick reconstitution capabilities, is particularly effective for encapsulating products like vaccines and antibodies that require rapid administration [25].

Recent advancements in material science, such as the development of novel scaffold fabrication methods like those proposed by Ilaria Silvestro et al., incorporate techniques like thermally induced phase separation enhanced by freeze-gelation and photo cross-linking. These innovative methods avoid chemical cross-linkers and allow precise control over porosity, which is crucial for applications in tissue engineering. Claire M. Brougham et al. further demonstrate the potential of freeze-drying in creating complex scaffold geometries for biomedical applications, showcasing a novel method to produce a collagen-based, heart valve-shaped scaffold with controlled porosity. These cutting-edge techniques are at the forefront of our research, promising exciting new possibilities in microencapsulation [28,29].

**Figure 3:**
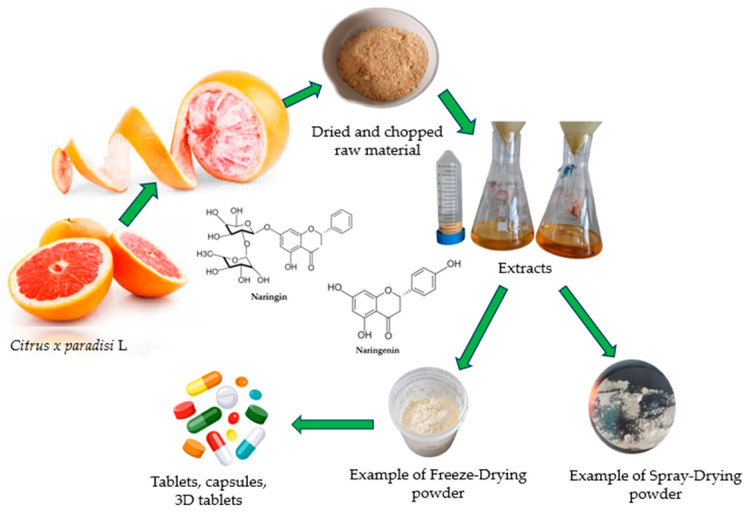
Converting grapefruit peels (*Citrus x paradisi.* L)into different pharmaceutical forms. The process starts with preparing the raw material by drying and chopping it, and then extracting the active compounds naringin and naringenin. Next, the emulsion is processed into powders using freeze-drying and spray-drying techniques.

This study aims to deepen the understanding of wall material selection for encapsulating bioactive compounds in grapefruit extract using both spray-drying and freeze-drying methods. By evaluating the roles of skim milk, maltodextrin, carboxymethylcellulose, and β-cyclodextrin as encapsulating agents, this investigation seeks to guide effective strategies for microencapsulation, focusing on the enhancement of powder quality and encapsulation efficiency Figure 3.

## 2. Materials and Methods

### 2.1. Citrus x paradisi.L Extracts Preparation

For these studies, we utilized the residual peels of *Citrus x paradisi* L., which are typically discarded post-juice-extraction. These peels were dried and ground into powder using a coffee grinder. The powdered peels underwent ultrasonic extraction in a Grant Instruments™ XUB12 Digital bath at 38 kHz with a 50% ethanol solution in a 1:10 ratio for 30 min at 50 ± 2 °C. After cooling to room temperature, the mixture was centrifuged at 1789× *g* for 10 min. The supernatant was filtered through a 0.22 µm PVDF filter for further microencapsulation.

### 2.2. Formulation of Emulsion for Spray-Drying and Freeze-Drying Processes

The wall material for spray- and freeze-drying comprised varying concentrations of maltodextrin, skim milk, beta-cyclodextrin, chitosan, and carboxymethylcellulose (CMC). The wall material, forming a 20% (*w*/*v*) concentration, was initially dissolved in purified water. Subsequently, all solutions were stirred using a magnetic stirrer (MSH-20A, Witeg, Wertheim, Germany) for 30 min at 25 °C. The solutions containing dissolved encapsulants were combined with a 50 mL ethanolic extract from *Citrus x paradisi* L. The resulting emulsion was stirred for another 30 min at 25 °C. Directly following this process, the resultant emulsion was used for microencapsulation purposes.

### 2.3. Parameters for Spray-Drying Process

Spray-drying was carried out using a Buchi B-291 Mini Spray-Dryer (BÜCHI Labortechnik AG, Flawil, Switzerland) under varying experimental conditions. These included inlet temperatures of 90, 120, and 160 °C, outlet temperatures ranging from 25 to 80 °C, a spray flow feed rate of 30 mL/min, and air pressure in the 8-bar range. The resulting spray-dried powders were collected and stored in a refrigerator at +4–7 °C to prevent avoidable changes in material properties.

### 2.4. Freeze-Drying Procedure

The prepared mixtures were initially subjected to freezing using a FORMA™ 88,000 Series laboratory freezer (Therma Scientific, Waltham, MA, USA) at a temperature of −80 °C for 24 h. Later, the frozen samples underwent lyophilization in a LyoQuest Telstar laboratory freeze-dryer (Wertheim, Germany) operating at −50 °C and 0.05 bar for 24 h. The resultant powders were collected, securely sealed in foil bags, and stored in a desiccator to ensure preservation and stability until further analysis.

### 2.5. Characterizations of the Microcapsules

The powders produced by spray-drying and freeze-drying were analyzed in detail, including moisture content, wettability, solubility, bulk and tapped volumes, product yield, encapsulation efficiency, and morphology.

#### 2.5.1. Determination of Moisture Content

The moisture content of the powder is determined by oven-drying the sample at 105 °C until it reaches a constant weight. The heating rate is approximately 0.11 °C per minute. The weight loss observed during this process, quantified as a percentage, accurately reflects the product’s moisture content [30]. These tests were performed in triplicate, and results were reported as the mean ± (*n* = 3).

#### 2.5.2. Wettability Analysis of Spray-Dried and Freeze-Dried Powders

Wettability for both spray-dried powder and freeze-dried powder was evaluated using methods adapted from Zhang et al. [31] and Wang et al. [32], respectively. In each test, 1 g of the sampler was added to 100 mL of water at room temperature, and the time until complete dissolution or disappearance from the water’s surface was recorded. These tests were performed in triplicate, and results were reported as the mean ± (*n* = 3).

#### 2.5.3. SEM Analysis of Microcapsules: Morphological Evaluation

The morphological properties of the microcapsules, as formulated using various wall materials and processed at 160 °C, were examined using scanning electron microscopy (SEM). Tiny quantities of the spray-dried powders were adhered to the surface of double-sided tape attached to stubs. The Hitachi TM 3000 scanning electron microscope, sourced from Tokyo, Japan, was employed to capture photomicrographs at magnifications ranging from 100× to 5000× under an accelerating voltage of 5 kV.

#### 2.5.4. Assessment of Process Yield (Y%)

For this study, the yield of each production process was calculated based on the amount of solid material initially introduced into the equipment compared to the quantity of powder collected after the process. The yield percentage was determined using the following adapted equation, Equation (1):

(1) $$\mathrm{Yield}\;(\%)=\frac{Weight\;of\;Powder\;Collected}{Weight\;of\;Solids\;Fed\;into\;Equipment}\;\times 100$$

#### 2.5.5. Measurement of Bulk and Tapped Volumes for Spray-Dried and Freeze-Dried Powders

The evaluations of bulk (*V*_0_) and tapped volumes (Vtapped) of both spray-dried and freeze-dried powders were conducted using the SVM 102 Erweka (Germany) density tester, following the protocols outlined in the Pharmacopeia (Ph. Eur., USP). These collected volume measurements were then applied to calculate each powder variant’s Carr index (2) and Hausner ratio (3). The tapped volume was determined after performing 750 tappings. These tests were performed in triplicate, and results were reported as the mean ± (*n* = 3).

(2) $$\mathrm{CarrIndex}=\frac{100\times (V_{0}-Vtapped)}{V_{0}}$$

(3) $$\mathrm{HausnerRatio}=\frac{V_{0}}{Vtapped}$$

#### 2.5.6. Solubility Assessments

The solubility of the samples was assessed using a modification of the method described in [33]. A 0.5 g sample was mixed with 12.5 mL of distilled water and stirred with a magnetic stirrer at 350 rpm at 25 °C for 30 min. The solution was centrifuged at 3500× *g* for 10 min at the same temperature. A 10 mL portion of the supernatant was dried overnight at 105 ± 5 °C. The weight difference determined the solubility percentage (%). These tests were performed in triplicate, and results were reported as the mean ± (*n* = 3).

(4) $$\mathrm{Solubility}\;(\%)=\frac{Residue\;after\;drying}{Theoretical\;residue\;after\;drying}\;\times 100$$

(5) $$\mathrm{Theoretical}\mathrm{residue}=\frac{W\;(supernatant\;to\;be\;dried)+W\;(microcapsules)}{W\;(microcapsules)-W\;(purified\;water)}\;\times 100$$

#### 2.5.7. Quantification of Total and Surface Phenolic Content in Powdered Samples

To find the total phenolic content (TPC) and surface phenolic content (SPC) of the powdered samples, an assay was made following a modified version of the method described in Pudziuvelyte et al. [6,25]. For the TPC determination, a 100 mg sample of the test powder was dissolved in a 1 mL solution of ethanol, acetic acid, and water, as mixed in a volume ratio of 20:8:42, respectively. This mixture was stirred for 2 min with a magnetic stirrer and then subjected to an ultrasonic bath for 20 min at 30 °C. After sonication, the mixture was filtered through a microfilter with a 0.45 μm pore size. A quantity of 100 μL of the filtered sample was mixed with 2.5 mL of Folin–Ciocalteu reagent and left in a dark place for 5 min. Following this incubation, 2 mL of a 7.5% Na2CO3 solution was added to the mixture, which was then thoroughly mixed and left in the dark for an additional hour at 25 °C. The TPC was quantified by measuring the absorbance at 760 nm using a UV/VIS 1800 Shimadzu spectrophotometer, with results expressed in mg of gallic-acid-equivalent per gram of powder.

To assess the SPC, another 100 mg sample of the test powder was mixed with 10 mL of ethanol–methanol solution in a 1:1 volume ratio and filtered similarly.

The encapsulation efficiency TPC EE% (7) and SPC% (6) were calculated using the following specific formulas:

(6) $$\mathrm{SPC}\;\%=\frac{surface\;phenolic\;compounds\;}{total\;phenolic\;compounds}\;\times 100$$

*TPC EE* (%) = 100 *− SPC* (%) (7)

## 3. Results and Discussion

### 3.1. Influences of Different Conditions of Microencapsulation on the Physicochemical Properties

A study was conducted, beginning with the creation of an extract from *Citrus x paradisi.* L fruit peels using 50% *v*/*v* ethanol. The extract was used to make encapsulated powders. The process involved drying and grinding the peels, followed by extraction, centrifugation, and filtration. (Section 2). The resulting flavanone extracts were used for encapsulation.

First, the properties of spray-dried powders were determined using a consistent composition of 10% skim milk and 10% maltodextrin to establish optimal conditions for microencapsulation. Four samples (M1, M2, M3, and M7) were prepared under this formulation, and the effects of temperature on the yield and moisture content of the powders were evaluated. The results are summarized in Table 2, illustrating the variations in yield percentages and moisture levels under different conditions.

The study found that the yield range extended from 48% to approximately 53%, while the moisture content ranged from about 5.31% to 7.60%. The differences in yield and moisture content were attributed to the various conditions employed during the spray-drying process.

Based on these studies, the optimal conditions for obtaining the highest yield with the lowest moisture content were an inlet temperature of 160 °C and an outlet temperature of 80 °C. The yield achieved under these conditions was approximately 52.95 ± 2.64%. The moisture content was also the lowest, at about 5.97 ± 0.298%.

Meanwhile, when the inlet temperature was increased to 170 °C and the outlet temperature to 116 °C, the yield decreased to 48.0 ± 2.4%, while evincing a slightly lower moisture content of 5.31 ± 0.265%. This could be because of the increased temperature in the drying process. Higher temperatures may lead to the degradation of phenolic compounds or the formation of impermeable skin around the microcapsules, which can trap moisture and reduce the yield.

### 3.2. Impact of Wall Material Composition on the Physicochemical Characteristics of Microcapsules

During the initial phase of the study, different wall materials were selected for evaluation, including maltodextrin (MD), skim milk (SK), β-cyclodextrin (β-CD), and carboxymethylcellulose (CMC). These materials were used to understand the physical and chemical properties of the resulting powders, in addition to the release characteristics of the powders. The concentration of the encapsulating agents was set at 20%. The microencapsulation conditions were optimized based on the study’s parameters as reported in Section 2.1.

**Figure 4:**
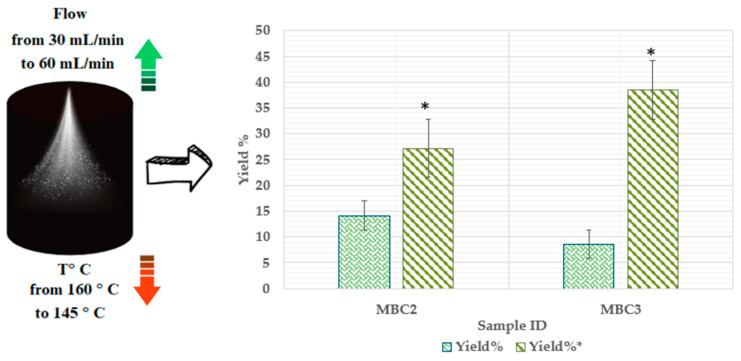
Illustration of how temperature and flow affect bulk quantities (Yield%). MBC2 * (MD 15%, β-CD 4.1%, and CMC 0.9%) *p* < 0.05 MBC2 (MD 15%, β-CD 4.1%, and CMC 0.9%); MBC3 * (MC 17%, β-CD 2.5%, and CMC 0.5%) *p* < 0.05 MBC3 (MC 17%, β-CD 2.5%, and CMC 0.5%).

Using different wall materials significantly impacts the parameters of the spray-drying process. For example, experiments using samples MBC2 and MBC3, which had varying amounts of MD, β-CD, and CMC under identical conditions, resulted in reduced quantities. However, improvements in yield were observed by modifying the temperature and flow rate. The temperature was decreased from 160 °C to 145 °C and the flow rate increased from 30 mL/min to 60 mL/min. These findings highlight the importance of both wall materials and operational parameters in determining the efficiency and result of the spray-drying process. The graph in Figure 4 illustrates how temperature and flow affect bulk quantities.

It was observed that yield improvements occurred for two bulk compositions, MBC2 and MBC3, when the temperature was reduced and the flow rate increased. The yield of MBC2 increased from 14.15% to 27.2%, while MBC3’s yield increased significantly, from 8.55% to 38.5%. This improvement is attributed to the lower temperature, which enhances solubility and stability. At the same time, the higher flow rate promotes better mixing and mass transfer. The specific composition of MBC3 was found to be more responsive to the process changes, resulting in the most significant yield increase.

#### 3.2.1. Examining Moisture Content and Wettability in Microcapsule Formulations

In a previous study, we determined suitable spray-drying parameters for microcapsules’ qualitative aspects. This study aims to examine how different wall materials, processed with the same parameters, affect moisture content and wettability.

**Figure 5:**
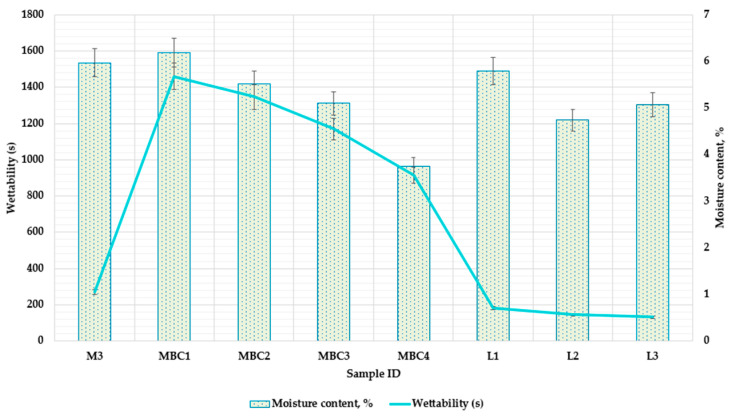
Comparative moisture content and wettability time analysis across different microcapsule formulations. M3 (MD 10%, SK 10%); MBC1 (MD 13%, β-CD 5.8%, CMC 1.2%); MBC2 (MD 15%, β-CD 4.1%, CMC 0.9%); MBC3 (MD 17%, β-CD 2.5%, CMC 0.5%); MBC4 (MD 10%, SK 9%, β-CD 1%). L1 (MD 13%, β-CD 5.8%, CMC 1.2%); L2 (MD 15%, β-CD 4.1%, CMC 0.9%); L3 MBC3 (MD 17%, β-CD 2.5%, CMC 0.5%); M indicates the spray-dry method, and the L–lyophilization method is used.

Figure 5 illustrates the variations in moisture content and wettability among different microcapsule formulations that employ distinct combinations of encapsulating agents. For instance, M3, which utilizes a balance of maltodextrin and skim milk, exhibited a moderate moisture content of 5.97 ± 0.29%. MD is recognized for its ability to confer low moisture content, which is advantageous for the stability of microcapsules over time, as supported by references [34,35]. Our findings indicate that increased maltodextrin correlates with decreased moisture levels, as demonstrated by MBC2 and MBC3 (5.52 ± 0.276% and 5.1 ± 0.255%, respectively), under consistent spray-drying conditions.

The integration of β-CD in formulations of MBC1, MBC2, and MBC3 seems to impact the surface characteristics of the microcapsules, leading to increased wettability times as the concentration of β-CD rises (Figure 5). This alteration may be due to the formation of more-structured and less-permeable surfaces, which is attributed to the β-CD [36]. β-CD is a cyclic oligosaccharide that can create complexes with various hydrophobic compounds within its structure while its exterior remains hydrophilic. This means that the surface of β-CD is predominantly hydrophilic, enhancing the wettability of microcapsules. However, the hydrophilic/hydrophobic properties of β-CD can be altered when other components are present in the composition of the microcapsules [37].

Carboxymethylcellulose (CMC), due to its hydrophilic properties, significantly influences the moisture content and wettability times of microcapsules, which are crucial for controlled release mechanisms [38]. In the MBC series, we observe that the wettability time decreases with decreasing CMC content: MBC1 (1.2% CMC) has a wettability time of 1461 s, MBC2 (0.9% CMC) 1347 s, and MBC3 (0.5% CMC) 1170 s.

Meanwhile, the sample MBC4 exhibited a lower moisture content and wettability time (3.75 ± 0.018% and 915 s, respectively), suggesting a synergistic interaction between SK and β-CD. This combination, involving the protein matrix and the encapsulating function of β-CD, results in a less hygroscopic product, and one which is more rapidly wettable [37].

Samples L1, L2, and L3 showcase the benefits of lyophilization in producing microcapsules with lower moisture content (5.8 ± 0.29%, 4.74 ± 0.19%, and 5.07 ± 0.25%). Freeze-drying typically produces a more porous structure that retains less moisture than those produced with spray-drying [15]. The rapid wettability of L2 (despite having a composition like L1) indicates that optimizing the ratio of MD to β-CD and CMC is crucial for improving water uptake. It suggests that the specific proportions of these components are critical to the microcapsules’ properties, beyond just the overall concentration of wall materials. L3 displayed a moisture content comparable to MBC3 but lower wettability due to the different drying methods.

#### 3.2.2. Impacts of Composition and Drying Methods on the Flowability of Microencapsulated Powders

**Figure 6:**
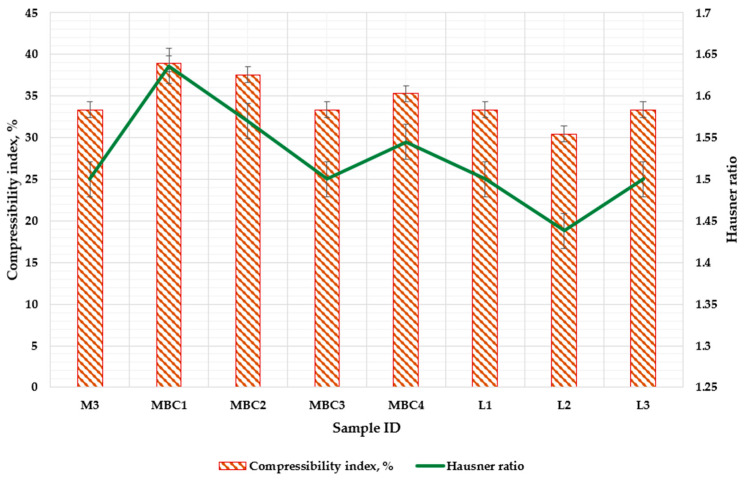
Assessment of compressibility (Carr index) and flowability (Hausner ratio) in spray-dried and freeze-dried powders formulated with varied wall materials. M3 (MD 10%, SK 10%); MBC1 (MD 13%, β-CD 5.8%, CMC 1.2%); MBC2 (MD 15%, β-CD 4.1%, CMC 0.9%); MBC3 (MD 17%, β-CD 2.5%, CMC 0.5%); MBC4 (MD 10%, SK 9%, β-CD 1%). L1 (MD 13%, β-CD 5.8%, CMC 1.2%); L2 (MD 15%, β-CD 4.1%, CMC 0.9%); L3 MBC3 (MD 17%, β-CD 2.5%, CMC 0.5%). M indicates the spray-dry method, and the L–lyophilization method is used.

The compressibility index and Hausner ratio are essential for assessing the powder characteristics of spray-dried and freeze-dried formulations. The compressibility index reflects the ability of a powder to settle and the degree to which it can be compacted [39]. The powder flowability, characterized by the Carr index and Hausner ratio, ranged from 30.43% to 38.89% and 1.438 to 1.636, respectively ( Figure 6). Based on the European Pharmacopoeia article Ph. Eur. 01/2010:20936, the flowability of the powders is classified from ‘poor’ to ‘very, very poor’ based on these measurements.

Mainly, the MBC1 sample exhibits a Hausner ratio of 1.636, one of the highest, indicating lower flowability. This is possibly due to its higher β-CD and CMC content, substances that tend to enhance cross-linking, which could diminish flow.

Conversely, incorporating more maltodextrins tends to lower the cohesiveness, thereby improving flow. This is demonstrated by the MBC3 sample, which has an increased MD content and reflects this principle.

SK is part of the microencapsulation process, as evidenced by samples like M3 and MBC4 (1.5–1.545 and 31.47% and 30.11%). The proteins in SK can enhance the integrity of the powder through protein–protein interactions, which may reflect increased compressibility indices and Hausner ratios, suggesting a reduction in flow. Nevertheless, statistical analyses have not found significant differences between groups of samples, suggesting that skim milk’s inclusion does not significantly impact flowability or compressibility when compared to the influence of other components.

The preparation method also plays a significant role in these properties. Although spray-drying typically results in more uniform and spherical particles, which should theoretically improve flow compared to the irregular particles from lyophilization, the composition’s influence is more pronounced than that of the drying technique. However, interestingly, lyophilized samples have shown better compressibility indices and Hausner ratios in cases where the compositions are similar, such as with MBC1 and L1.

#### 3.2.3. Optimization of Solubility and Release Profiles in Microencapsulated Phenolic Compounds

**Figure 7:**
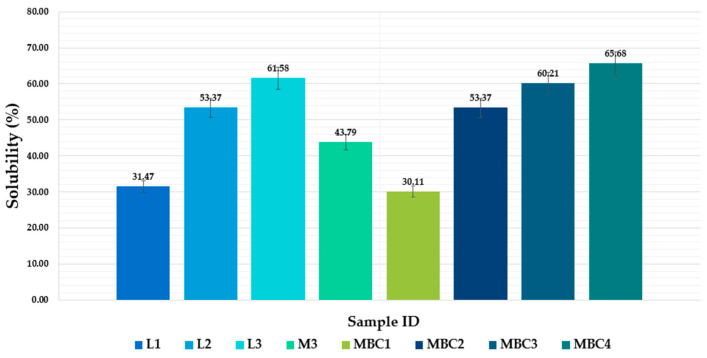
Solubility (%) of microencapsulated formulations using spray-drying and lyophilization methods with a different wall material. M3 (MD 10%, SK 10%); MBC1 (MD 13%, β-CD 5.8%, CMC 1.2%); MBC2 (MD 15%, β-CD 4.1%, CMC 0.9%); MBC3 (MD 17%, β-CD 2.5%, CMC 0.5%); MBC4 (MD 10%, SK 9%, β-CD 1%). L1 (MD 13%, β-CD 5.8%, CMC 1.2%); L2 (MD 15%, β-CD 4.1%, CMC 0.9%); L3 MBC3 (MD 17%, β-CD 2.5%, CMC 0.5%). M indicates the spray-dry method, and the L–lyophilization method is used.

During our research, we observed significant variations in the solubility of different samples, indicating that the choices of composition and preparation method significantly impact their solubility. The samples were prepared using lyophilization (L1, L2, and L3) and spray-drying (M3, MBC1, MBC2, MBC3, and MBC4). Figure 7 displays the solubility percentages of microencapsulated formulations using spray-drying and lyophilization methods with different wall materials.

Samples prepared using the lyophilization method showed an increasing tendency towards increased solubility with a decrease in β-CD and CMC content and an increase in MD content. The solubility ranged from 31.47 ± 1.57% for L1 to 61.58 ± 3.079% for L3. We suggest that MD, a polysaccharide, can increase the solubility of samples, while β-CD and CMC may decrease it [40].

A similar tendency can be observed for samples prepared using the spray-dry method. Solubility increased with the decrease in β-CD and CMC content and an increase in MD content. The solubility ranged from 30.11 ± 1.50% for MBC1 to 65.68 ± 3.35% for MBC4.

Lyophilization produces formulations with a more porous structure, which could lead to faster release rates [15]. Nonetheless, our data indicate that with a careful selection of wall materials, even lyophilized samples can achieve a degree of solubility suitable for controlled release.

Spray-drying is known for producing denser particles, which should, in theory, contribute to a slower release due to reduced solubility [35]. Based on our research data for the spray-dried samples (marked as M), the MBC3 sample is the most suitable for producing modified-release microcapsules to encapsulate grapefruit phenolic compounds. This sample has a wettability time of 1170 s, indicating a potentially slower release rate, which is desirable for sustained release. Additionally, its moisture content is 5.1% and it has a high solubility of 67.05%. These factors make it beneficial for controlled release and bioavailability.

In the lyophilized (freeze-dried) sample series (marked as L), the L2 sample appears to be the most suitable choice. This sample has a relatively low moisture content of 4.74%, suggesting improved stability. It also exhibits excellent flowability, with a Hausner ratio of 1.438, which is crucial for manufacturing processes. Furthermore, its solubility is relatively high at 60.21%, and with a wettability time of 144 s, it might provide a rapid initial release while maintaining a controlled release profile.

#### 3.2.4. The Impact of Wall Material Composition on the Encapsulation Efficiency of Active Ingredients

**Figure 8:**
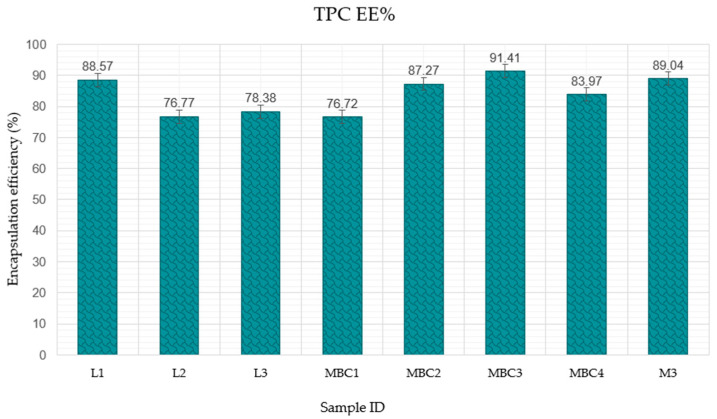
Encapsulation efficiency of *Citrus x paradisi.* L phenolic compounds in various microencapsulation formulations. M3 (MD 10%, SK 10%); MBC1 (MD 13%, β-CD 5.8%, CMC 1.2%); MBC2 (MD 15%, β-CD 4.1%, CMC 0.9%); MBC3 (MD 17%, β-CD 2.5%, CMC 0.5%); MBC4 (MD 10%, SK 9%, β-CD 1%). L1 (MD 13%, β-CD 5.8%, CMC 1.2%); L2 (MD 15%, β-CD 4.1%, CMC 0.9%); L3 MBC3 (MD 17%, β-CD 2.5%, CMC 0.5%).

In examining the M and L series’ encapsulation efficiency (EE%), sample MBC3 from the M series achieves the highest encapsulation efficiency, at 91.41%. Figure 8 illustrates the encapsulation efficiency percentages (TPC EE%) for various microencapsulated formulations. M3 with equal parts MD and SK (both at 10%) shows a high EE% at 89.04, suggesting that combining these two components at these specific ratios is conducive to effective encapsulation. In comparison, MBC1 has the lowest value within this group, at 76.72%. These results indicate that the optimal ratio of maltodextrin, as observed in MBC3, is crucial for enhancing encapsulation efficiency. EE% increases recorded with increasing MD in the formulation M sample were 76.72% < 87.27% < 91.41%. MBC4, with a high proportion of SK comparable to MD and a minimal amount of β-CD, shows an EE% of 83.97, which is lower than M3 but higher than MBC1. The presence of SK at a high level seems beneficial but the impact is not as pronounced as that of a high MD content. L1 is the highest for the L series, at 88.57%, with L2 at the lower end at 76.77%. However, the L series overall exhibits lower encapsulation efficiencies than their MBC counterparts.

#### 3.2.5. Scanning Electron Microscopy of Spray-Dried and Freeze-Dried Powders

**Figure 9:**
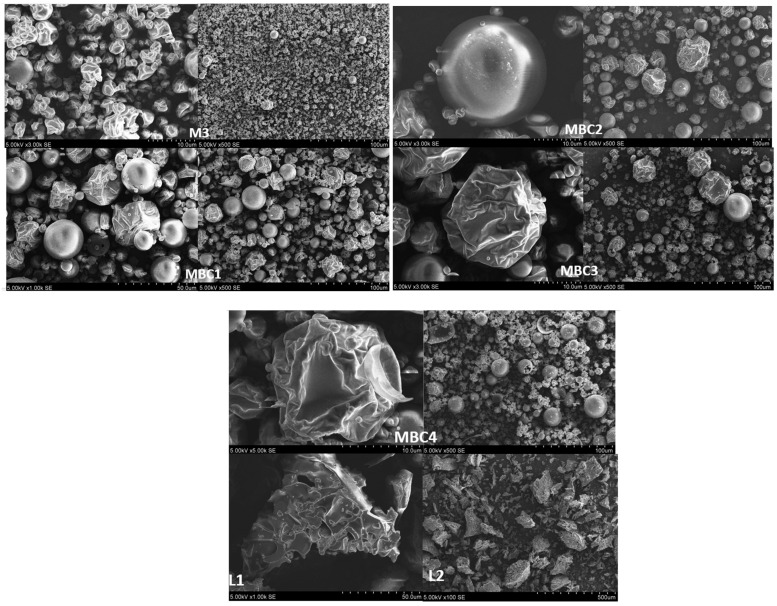
Microcapsules were observed using scanning electron microscopy (SEM) to capture their morphology at 100×, 500×, 1000×, 3000×, and 5000× magnifications. M3 (MD 10%, SK 10%); MBC1 (MD 13%, β-CD 5.8%, CMC 1.2%); MBC2 (MD 15%, β-CD 4.1%, CMC 0.9%); MBC3 (MD 17%, β-CD 2.5%, CMC 0.5%); MBC4 (MD 10%, SK 9%, β-CD 1%). L1 (MD 13%, β-CD 5.8%, CMC 1.2%); L2 (MD 15%, β-CD 4.1%, CMC 0.9%).

Figure 9 presents scanning electron microscope (SEM) images of various microencapsulated formulations identified as M3, MBC1, MBC2, MBC3, MBC4, L1, and L2. The images show the surface morphology and particle size distribution of each sample at high magnifications, providing insight into the physical characteristics of the microcapsules produced by different methods and with various wall materials. Each panel highlights unique structural differences, from spherical and smooth to irregular and crumpled textures, which are critical in understanding the encapsulation efficiencies and potential release behaviors of the encapsulated compounds.

SEM images of M3 show semi-spherical microcapsules with a wrinkled surface, likely due to the combination of maltodextrin and skim milk. This texture could influence the microcapsules’ release profile and surface area, potentially enhancing their interaction with the environment. MBC1 (MD 13%, β-CD 5.8%, CMC 1.2%) combines smooth and wrinkled particles with a softer surface due to the higher β-CD content. MBC2 (MD 15%, β-CD 4.1%, CMC 0.9%) shows fewer wrinkles and a more uniform surface with increased MD and decreased β-CD and CMC content compared to MBC1. The MBC3 (MD 17%, β-CD 2.5%, CMC 0.5%) sample has the most uniform and smoothest surface morphology due to the high MD content. MBC4 (MD 10%, SK 9%, β-CD 1%), with a high proportion of skim milk, produces a unique texture, possibly showing a balance between smooth and wrinkled surfaces.

L1 (MD 13%, β-CD 5.8%, CMC 1.2%) and L2 (MD 15%, β-CD 4.1%, CMC 0.9%) samples were lyophilized, and they exhibited a more porous and irregular structure than those associated with spray-drying. SEM images provide crucial information on how the different wall material ratios influence the physical structure of the microcapsules, which in turn affects their functional properties. The micrographs reveal any agglomerations, cracks, or inconsistencies within the microcapsules that could impact their effectiveness.

## 4. Conclusions

In this investigation, the ethanolic extract of *Citrus x paradisi* L. was subjected to microencapsulation through spray drying and freeze-drying techniques, employing combinations of skim milk, maltodextrin, carboxymethylcellulose, and beta-cyclodextrin as wall materials. The chosen matrix components were necessary for forming microparticles and encapsulating the ethanolic extract, with each constituent playing a distinct role in the microcapsules’ structural and release properties.

Maltodextrin (MD), as a polysaccharide, was essential in the microcapsule’s structural formation, providing a protective matrix for the active compounds. Beta-cyclodextrin (β-CD), a cyclic oligosaccharide, was used to enhance the solubility and stability of hydrophobic molecules by forming inclusion complexes. This characteristic of β-CD was beneficial in improving the bioavailability of the active compounds. Carboxymethylcellulose (CMC), with its hydrophilic properties, acted as a release modulator within the microcapsules. The formation of hydrogels by CMC was essential in controlling the release rate of the encapsulated actives in the intestinal tract.

The comparative analysis of spray-dried and freeze-dried samples showed notable differences in morphologies and encapsulation efficiencies. In the spray-dried methodology, sample ID MBC3 (MD 17%, β-CD 2.5%, CMC 0.5%) showed the best results. It was characterized by its wettability time (1170 s), a higher encapsulation efficacy EE% (91.41%), a better solubility (60.21%), and lower moisture content (5.1 ± 0.255%), which can be attributed to the higher maltodextrin content, indicating a solid structure conducive to prolonged release. Meanwhile, the freeze-dried sample ID L1 (MD 13%, β-CD 5.8%, CMC 1.2%) displayed a quick weldability (132.6 s), rapid solubility profile (61.58%), low moisture content (5.07%) and high EE% (78.38%), which may result from the lyophilization process and the wall material composition, which facilitated a more porous structure.

This research contributes to the field of pharmaceutical sciences by illustrating the criticality analysis of wall material selection and process optimization in microencapsulation. It highlights the necessity for a multidisciplinary approach which considers material science and pharmacokinetics in order to develop advanced delivery systems for active bioactive compounds.

## Figures and Tables

**Table 1:** Encapsulation coating materials and their applications [10,11,12,13,14].

Microencapsulation Materials	Material Examples	Common Use
Polysaccharides	Dextrines (maltodextrin, cyclodextrins), Ethylcellulose, Methylcellulose, Hydroxypropyl methylcellulose, Carboxymethylcellulose, Carrageenan	Food, Pharmaceuticals, Nutraceuticals
Proteins	Gelatin, Casein, Whey protein, Skim milk, Egg white	Food, Pharmaceuticals, Nutraceuticals
Lipids	Waxes (beeswax, carnauba wax), Animal sources, Fats, and Plant sources	Food, Pharmaceuticals, Nutraceuticals
Synthetics	Poly-lactic-co-glycolic acid (PLGA)	Target drug delivery, Bioengineering

**Table 2:** Spray-drying conditions and microencapsulation results for *Citrus x paradisi.* L phenolics using maltodextrin (MD) 10% and skim milk (SK) 10% as wall materials (M1, M2, M3, and M7).

Inlet T (°C)	Outlet T (°C)	Flow Rate (mL/min)	Air Pressure	Yield (%)	Moisture Content (%)	Sample ID
90	25	30	8 bars	48.10 ± 2.40	7.60 ± 0.38	M1
120	65	30	8 bars	51.65 ± 2.58	6.57 ± 0.32	M2
160	80	30	8 bars	52.95 ± 2.64	5.97 ± 0.298	M3
170	116	30	8 bars	48.00 ± 2.40	5.31 ± 0.265	M7

## Data Availability

Data is contained within the article.
